# The French Version of the Reflective Functioning Questionnaire: Validity Data for Adolescents and Adults and Its Association with Non-Suicidal Self-Injury

**DOI:** 10.1371/journal.pone.0145892

**Published:** 2015-12-29

**Authors:** Deborah Badoud, Patrick Luyten, Eduardo Fonseca-Pedrero, Stephan Eliez, Peter Fonagy, Martin Debbané

**Affiliations:** 1 Developmental Clinical Psychology Unit, Faculty of Psychology, University of Geneva, Geneva, Switzerland; 2 Office Médico-Pédagogique Research Unit, Department of Psychiatry, University of Geneva School of Medicine, Geneva, Switzerland; 3 Research Department of Clinical, Educational and Health Psychology, University College London, London, United Kingdom; 4 Faculty of Psychology and Educational Sciences, KU Leuven, Leuven, Belgium; 5 Department of Educational Sciences, University of La Rioja, La Rioja, Spain; 6 Center for Biomedical Research in the Mental Health Network (CIBERSAM), Madrid, Spain; University of Vienna, School of Psychology, AUSTRIA

## Abstract

**Introduction:**

The capacity to understand one’s own actions and those of others in terms of cognitive and affective mental states (i.e., reflective functioning or mentalizing) is thought to play a critical role in both typical and atypical development. To date, however, no self-report tool is available for assessing reflective functioning ability in French-speaking samples. The first aim of this study is to investigate the reliability and validity of the reflective functioning questionnaire (RFQ) in French-speaking adolescents and adults. Secondly, we investigate whether low levels of reflective functioning were associated with non-suicidal self-injury.

**Methods:**

130 adolescents (66 females, *M*_age_ = 15.72, *SD*_age_ = 1.74) and 253 adults (168 females, *M*_age_ = 23.10, *SD*_age_ = 2.56) completed a French translation of the RFQ and a battery of self-reported questionnaires to assess a set of clinical (alexithymia; borderline traits; internalizing and externalizing symptoms) and psychological (empathy; mindfulness) variables.

**Results:**

The current results showed configural invariance of the original two-factor structure of the RFQ across French-speaking adolescents and adults and satisfactory reliability and construct validity of the two subscales. Furthermore, we observed that recent episodes of non-suicidal self-injury were associated with lower levels of reflective functioning in the adult, but not in the adolescent, sample.

**Discussion:**

The present research has methodological and clinical implications in that it provides the first evidence that the RFQ can be used to reliably assess reflective functioning in French-speaking population. The study further shows that impaired ability to consider mental states that lie behind behaviors might play a role in non-suicidal self-injury, at least in adults.

## Introduction

Reflective functioning (RF) refers to the empirically grounded operationalization of psychological processes that sustain the capacity to mentalize, that is, to understand and interpret behaviors of the self and others as expressions of intentional mental states such as beliefs, desires, or needs [1]. Theoretically, RF emerges from the convergence of two theories, so-called social biofeedback theory [2] and the development of psychic reality theory [3]. The social biofeedback theory describes how, through the process of parental affect mirroring, primary attachment relationships constitute the “breeding ground” for emotional self-awareness and the development of self-control in infancy. The second theory relates to how children perceive psychic reality and progressively move from experiencing inner and outer reality as either equivalent (psychic equivalence) or dissociated (pretend) toward a more integrated, reflective mode.

Empirically, data have been collected from three strands of research on RF. A first line of studies focusing on the impact of parental RF on child development has suggested that RF might constitute the missing link in the often-cited intergenerational transmission of attachment between parent and infant [4–5]. Parental RF may also contribute to adolescents’ RF and social competencies [6]. Alongside these studies on parental RF, a second wave of studies has investigated individual differences in reflective abilities and identified disrupted RF processes in adults with various psychiatric conditions, including psychosis, depression, borderline personality disorder (BPD), panic disorder, and eating disorders (see [7–8] for reviews). More recently, impairments in RF have also been related to borderline trait levels in adolescents [9–12]. This evidence has prompted a third wave of studies, which have focused on the role of RF in clinical settings and explored the influence of RF in psychotherapeutic processes and outcomes (e.g., [13–16]).

Together, the findings of these three waves of studies bring to light the potential pivotal role of RF in typical and atypical development, as well as in psychotherapeutic processes and outcomes. While the data from existing research are valuable, some aspects of RF remain largely unstudied.

On the one hand, with respect to dysfunctions of RF, results are mainly biased toward clinical samples, and there is little evidence for the role of RF in healthy samples. Reports addressing associations between the level of RF and specific maladaptive behaviors (rather than defined psychiatric syndromes) in non-referred youths are, to date, rare and have produced inconclusive findings [7]. Community-based empirical designs are not only essential for the development of early identification and prevention strategies; at the same time they sidestep the problem of potential confounding factors (e.g. medication, effects of enduring pathology and multiple treatments, comorbidity, etc.). Non-Suicidal Self-injury (NSSI) represents a critical target for investigation because it is a frequent problem among adolescents and young adults (see [17] for a meta-analysis) and is linked to a range of clinical correlates (e.g., [18]). Notably, the presence of NSSI has been related to the severity of borderline personality features. On the basis of findings reporting that 30% of adults with BPD retrospectively reported the onset of NSSI in adolescence [19], some authors consider self-harming behaviors to be a potential adolescent precursor of BPD [20]. In view of the results of several studies linking dysfunctions in reflective capacity to BPD or borderline traits in both adulthood and adolescence, it can therefore be expected that impairments in RF are also associated with NSSI.

On the other hand, and perhaps more significantly with regard to research progress, current conclusions are restricted by measurement concerns. A substantial part of the data has been collected using the RF Scale [21], which rates the capacity to reflect on attachment relationships using transcripts of the Adult Attachment Interview [22] and the Parent Development Interview. Use of the RF scale in an empirical context is limited by issues of practicality. Moreover, the duration and cost of this clinical interview mean that it is not feasible to use it in most clinical and research settings (e.g., [9]).

Based on the adult version, an RF scale for youths [23] has recently been developed. This scale has been shown to display adequate reliability and validity [24]. However, like its adult counterpart, it is expensive and time-consuming. In trying to bypass these limitations, another set of studies has instead used validated self-report questionnaires measuring constructs that theoretically overlap with RF, such as empathy, mindfulness or, metacognition [25]. Although these measures are somewhat more feasible and statistically reliable, they are limited by the fact that they provide only a partial and indirect assessment of RF and emanate from other theoretical backgrounds.

In order to address these issues, Fonagy and collaborators [26] have recently created a new instrument, the Reflective Function Questionnaire (RFQ), which is specifically designed for RF assessment. The first version of this self-report questionnaire (RFQ46) included 46 items and was piloted for reliability and validity in clinical and nonclinical adult samples [26–28]. Ha et al. [9] tested a version of the original RFQ46 adapted for use with youths (RFQY46) in an inpatient adolescent sample. The study demonstrated satisfactory internal consistency and highlighted expected correlations between the total score on the RFQY46 and experimental and interview-based assessments of RF. In addition, a low RFQY46 total score was related to high borderline personality features, consistent with adult studies that have established the presence of poor RF in individuals with BPD (e.g. [26, 29]).

Further research with this instrument led to the identification of two subscales in the RFQ assessing Certainty (RFQc) and Uncertainty (RFQu) about the mental states of self and others [26]. Extreme scores on these two subscales reflect two impairments in reflective functioning that are common in many mental disorders, i.e., hypermentalizing and hypomentalizing respectively. Hypermentalizing refers to a tendency to develop excessively detailed models of the mind of oneself that go far beyond the available evidence. Hypomentalizing, by contrast, reflects concrete thinking characterized by an absence or unwillingness to develop nuanced, more complex models of the mind of others and/or the self. Considering the valuable empirical work that has so far dealt with issues surrounding the assessment of RF, the aims of the present study are two-fold. Since the RFQ was developed in English and is not currently available for French-speaking individuals, our first purpose was to translate and validate the RFQ.

Specifically, we expected to find evidence for a two-factor structure, referring respectively to the degree of subjective confidence or doubt that actions are mentally driven. We furthermore assumed that this two-factor structure was invariant across adolescent and adult samples, and that both scales would have satisfactory internal consistency. In addition, correlations between RFQ subscale scores and clinical variables (levels of general psychopathological symptoms and borderline traits), as well as psychological capacities (mindfulness, empathy, alexithymia) that have been previously linked with RF both theoretically and empirically, were examined. We hypothesized that there would be *positive* associations between the degree of certainty concerning mental states (RFQc) and both a) mindfulness dimensions of acting with awareness and description of internal states and b) levels of cognitive empathy. Conversely, we expect to find *negative* relationships between the degree of certainty concerning mental states (RFQc) and a) alexithymia dimensions regarding the extent to which thoughts are externally focused, and difficulties in identifying and describing feelings; b) borderline traits; and c) levels of internalizing and externalizing psychopathology. We expect to observe a reverse pattern of associations between the factors listed above and the degree of uncertainty about mental states (RFQu).

Finally, we evaluated whether between-subject differences in total RF score were associated with recent episodes of NSSI in a non-referred sample. To our knowledge no study to date has directly addressed the relationships between level of RF and presence of NSSI. Nevertheless, a large body of work relates impairments in RF to adult and adolescent BPD and borderline traits ([30–31] for reviews). Thus, we expected individuals with low levels of RF to be more likely to report an episode of NSSI in the previous year than individuals whose level of RF was higher.

## Material and Methods

### Participants

Participants were community adolescents and young adults from Geneva, Switzerland, who were recruited via written advertisements and word of mouth. Inclusion criteria comprised age (at least 12 years of age), language (native French speakers) and, for adolescents (ages 12–18 years), parental consent. Research objectives were presented to each potential participant (and, for adolescents, the parents) and they then decided whether they wished to volunteer for the study. The final sample was divided by age into a group of 130 adolescents (66 female, aged 12–18 years old, *M*_age_ = 15.72, *SD*_age_ = 1.74) and a group of 253 adults (168 female, aged 19–31 years old, *M*_age_ = 23.10, *SD*_age_ = 2.56). Written informed consent was received from the participants (and the parents of adolescents if below than 18 years old) under protocols approved by the cantonal ethics committee for human research of Geneva and by the ethics committee of the psychology and educational sciences department of the University of Geneva.

### Measures

The following battery of self-report questionnaires was individually administered under the supervision of trained clinical psychologists (M.D. and D.B.) to make sure participants understood the items.

The Reflective Functioning Questionnaire (RFQ) [26] measures the level of certainty (RFQc) and uncertainty (RFQu) about mental states. The *Certainty about Mental States* (RFQc) subscale consists of 6 items focusing on the extent to which individuals disagree with statements such as “*I don’t always know why I do what I do*”. All items are scored by participants on a 7-point Likert type scale, ranging from “completely disagree” to “completely agree”. Items are subsequently rescored to capture more extreme levels of certainty, so that very low agreements on this scale reflect hypermentalizing, while some agreement reflects adaptive levels of certainty about mental states. To this end, these items are recoded to 3, 2, 1, 0, 0, 0, 0. The *Uncertainty about mental states* (RFQu) subscale, which in the extreme captures hypomentalizing, also consists of 6 items scored on the same 7-point Likert type scale. Responses to items such as “*Sometimes I do things without really knowing why*”, are recoded to 0, 0, 0, 0, 1, 2, 3, again to ensure that high scores reflect a stance characterized by an almost complete lack of knowledge about mental states, while lower scores reflect acknowledgment of the opaqueness of one’s own mental states and that of others, typical of genuine mentalizing.

The Cognitive subscale of the Basic Empathy Scale (BES) [32–33] was used to estimate participants’ level of cognitive empathy (BEScog). The BES includes nine items rated on a 5-point Likert scale ranging from “strongly disagree” to “strongly agree”.

The Toronto Alexithymia Scale (TAS) [34–36] encompasses 20 items (rated on a 5-point scale), yielding three subscale scores which measure difficulties in identifying feelings (TASif), difficulties in describing feelings (TASdf), and lack of focus on internal emotional experiences (external-oriented thinking; TASeot).

The Kentucky Inventory of Mindfulness Skills (KIMS) [37] is a 39 item inventory (rated on a 5-point scale) used to assess abilities in focusing one’s attention in a non-judgemental way or accepting the occurrence of a present experience. The Describing (KIMSdes) and Acting with awareness (KIMSac) subscales were used in this study.

The Borderline Personality Inventory (BPI) [38–39] was used to assess participants’ total level of borderline traits (BPItot) in the previous year, based on 54 items rated on a 7-point scale. Item 20 in the inventory (“I have already non-suicidally self-injured myself”) was dichotomized (1 = never, 2–7 = at least one episode) to identify participants who had recently engaged in NSSI.

The Youth and Adult Self-Reports (YSR/ASR) [40–41] measure the level of general internalizing (YSR/ASRint) and externalizing (YSR/ASRext) symptoms. Participants rated the extent to which a series of 119 statements described their behavior over the past 6 months, using a 3-point scale.

### Data analyses

The validation process of the French RFQ started with the translation of the original English RFQ by independent French and English native speakers, following a forward-backward-forward procedure [42]. Psychometric properties of the RFQ were assessed as follows. First we conducted a confirmatory factor analysis (CFA) for the proposed two-factor model in both samples. Then a configural invariance model was tested using multi-group CFA. The Weighted Least Squares Means and Variance adjusted (WLSMV) estimator was used [43]. The configural model is established by specifying and testing the CFA model for each group separately. Once the theoretical model was validated in both groups, configural invariance was examined. Configural invariance requires that the pattern of fixed and freely estimated model parameters are equivalent across groups [44]. It is worth noting that six Correlated Errors (CE) terms (or disturbances) were allowed. Indeed, while taking into account the problem of using CE in CFA framework [45], we based the choice of computing certain CE upon theoretical (i.e., measurement model provided by the authors of the original instrument, which include CE terms for the items with similar content) and as well as a statistical argument (i.e., high modification indices and large standardized residuals in Mplus). Thus, in this study, CE terms were used conservatively and based on a theoretical rationale.

The goodness-of-fit indices employed were: chi-square; the Comparative Fit Index (CFI), the Tucker-Lewis Index (TLI), the Root Mean Square Error of Approximation (RMSEA) (and 90% confidence interval), and the Weighted Root Mean Square Residual (WRMR). To achieve a good fit of the data, the values of CFI and TLI should be over 0.95, and the RMSEA values should be under 0.08 for a reasonable fit and under 0.05 for a good fit [46–47]. For the WRMR values, a value below 1.0 has been suggested as indicative of adequate model fit [48]. The reliability of the obtained subscales was estimated by calculating Cronbach’s alpha coefficients and mean inter-item correlation. Temporal stability was also estimated in a subsample of adults (*n* = 93) using test–retest correlations after a 3-week interval.

Construct validity was further established by examining the Spearman correlations between the RFQ subscales correlated with related constructs and indices of maladaptive psychological functioning. Regarding associations between the reflective functioning subscales and recent episodes of NSSI, two dichotomised group variables (high/low certainty and high/low uncertainty) were created on a median-split basis. Chi-squared tests and phi coefficients were calculated to measure the association between level of certainty and uncertainty about mental states (high versus low) and recent history of NSSI episode(s) (presence vs. absence). Bonferonni corrections for multiple correlations were applied in all these analyses.

## Results

### Measurement invariance of the RFQ scores across age

The hypothesized two-factor model showed an adequate to good fit to the data in each sample separately (see Table 1). Similarly, the configural invariance model in which no equality constraints were forced exhibited adequate fit to data. All the standardized coefficients for the configural two-factor model were statistically significant (*p* < .01) (see Table 2), except for one item in the adolescent group.

**Table 1:** Goodness-of-fit indices of the French Reflective Functioning Questionnaire across groups.

	χ^2^	*df*	CFI	TLI	RMSEA [90% CI]	WRMR
*Group*						
Adolescent (n = 123)	74.453	47	.962	.946	.069[.037-.098]	.726
Adult (n = 253)	66.184	47	.993	.990	.040[.012-.061]	.673
*Multi-Group*						
Configural Invariance	151.505	95	.983	.977	.056[.039-.073]	1.038

χ^2^: Chi Square; *df*: Degrees of freedom; RMSEA: Root Mean Square Error of Approximation; CI: Confidence Interval; CFI: Comparative Fit Index; TLI: Tucker-Lewis Index; WRMR: Weighted Root Mean Square Residual.

**Table 2:** Factor loading, factor covariance and error term covariance for measurement invariance across age

	Adolescent Group		Adult Group	
	Factor Loadings (SE)			
RFQitem	Factor 1	Factor 2	Factor 1	Factor 2
Cs1	.67(.07)		.73(.05)	
Cs16	.58 (.08)		.43(.06)	
Cs20	.55(.08)		.55(.07)	
Cs36	.93(.06)		.87(.04)	
Cs40	.60(.07)		.52(.06)	
Cs44	.39(.10)		.37(.07)	
Us16		.61(.09)		.75(.07)
Us36		.64(.07)		.53(.06)
Us40		.61(.14)		.50(.10)
Us44		.93(.07)		.84(.07)
Us28		.48(.09)		.53(.07)
Us8		.22(.12)		.37(.08)
	Factor covariance (SE)			
Factor1-Factor2	-.74 (.05)		-.76(.04)	
	Error term covariance (SE)			
Ecs40-Ecs16	.63(.08)		.70(.04)	
Eus40-Ecs20	-1.14(.07)		-1.09(.05)	
Eus36-Ecs16	-1.60(.05)		-1.08(.02)	
Eus36-Ecs40	-.57(.10)		-.62(.06)	
Eus16-Ecs1	-1.19(.07)		-1.05(.08)	
Eus44-Ecs36	-2.27(.09)		-1.64(.25)	

Cs stands for certainty scale items, Us for uncertain scale items, SE for Standard Error and, E for Error term.

All coefficients are statistically significant (*p* < .01), except the factor loading of US8 in the adolescent group (*p* = .06)

### Reliability

#### Adult group

Internal consistency was satisfactory for the RFQc subscale (Cronbach’s alpha = .719, mean inter-item correlation = .296), and slightly lower but still satisfactory for the RFQu subscale (Cronbach’s alpha = .644, mean inter-item correlation = .283). Moreover, the RFQ was administered a second time, three weeks after the initial administration, to 93 participants (68 females, *M*_age_ = 23.71, *SD*_age_ = 3.17). Correlations performed to measure temporal stability showed significant positive correlations between the first and second RFQ administrations (RFQc–RFQc retest: *r* = .701, *p* = .001; RFQu–RFQu retest: *r* = .545, *p* = .001; see Table 3).

**Table 3:** Reliability indices (internal consistency and temporal stability) for adolescent and adult groups.

		Reliability		
		Cronbach’s Alpha	Mean Interitem	Test-retest correlation
Adult Sample	RFQc	.719	.296	.701**
	RFQu	.644	.283	.545**
Adolescent Sample	RFQc	.739	.319	
	RFQu	.675	.257	

RFQc and RFQu stand for Reflective Functioning Questionnaire certainty and uncertainty

**p < .01

#### Adolescent group

Internal consistency was satisfactory for the RFQc subscale (Cronbach’s alpha = .739, mean inter-item correlation = .319) and for the RFQu subscale (Cronbach’s alpha = .675, mean inter-item correlation = .257; see Table 3).

### Validity indices of both subscales

#### Adult group

With regard to construct validity (Table 4), the RFQc subscale was positively correlated with both mindfulness dimensions (KIMSac *r* = .248, *p* = .00; KIMSdes *r* = .312, *p* = .001) and cognitive empathy (BEScog *r* = .311, *p* = .001); negative associations were found with all three alexithymia subscales (TASif *r* = -.455, *p* = .00; TASdf *r* = -.292, *p* = .001; TASeot *r* = -.245, *p* = .001), internalizing and externalizing psychopathology symptoms (ASRext *r* = -.464, *p* = .001; ASRint *r* = -.302, *p* = .001), and borderline traits (BPItot *r* = -.472, *p* = .001). In contrast, the RFQu subscale was negatively related to both mindfulness dimensions (KIMSac *r* = -.301, *p* = .001; KIMSdes *r* = -.285, *p* = .001) and BEScog (*r* = -.173, *p* = .001), while positive associations were found with all three dimensions of alexithymia (TASif *r* = .566, *p* = .001; TASdf *r* = .386, *p* = .001; TASeot *r* = .141, *p* = .001), internalizing and externalizing psychopathology symptoms (ASRext *r* = .527, *p* = .001; ASRint *rs* = .446, *p* = .001), and borderline traits (BPItot *r* = .487, *p* = .001).

**Table 4:** Construct validity indices for adolescent and adult groups.

	Adult Group		Adolescent Group	
	RFQc	RFQu	RFQc	RFQu
Y/ASRint	-.464**	.446**	-.287**	.218*
Y/ASRext	-.302**	.527**	-.261*	.349**
BPItot	-.472**	.487**	-.383**	.394**
TASif	-.455**	.566**	-.425**	.457**
TASdf	-.292**	.386**	-.334**	.351**
TASeot	-.245**	.141**	-.004	.030
BEScog	.311**	-.173	.203*	-.089
KIMSac	.248**	-.285**	.380**	-.313*
KIMSdes	.312**	-.301**	.228**	-.176

RFQc and RFQu stand for Reflective Functioning Questionnaire certainty and uncertainty; Y/ASR for Youth and Adult Self–Reports; BPI for Borderline Personality Inventory; TASif, df, eot for Toronto Alexithymia Scale, difficulties in identifying feelings, describing feelings and external oriented thinking; BEScog for Basic Empathy Scale cognitive; KIMSac, des for Kentucky Inventory of Mindfulness Skills Acting with awareness and Describing

* p < .01

** significant after Bonferonni corrections (*p* < .005).

All the correlations survived after Bonferroni corrections.

#### Adolescent group

The results for construct validity (Table 4) in the adolescent group largely mirror those for the adult sample. The RFQc was positively correlated with both mindfulness dimensions (KIMSac *r* = .380, *p* = .001; KIMSdes *r* = .228, *p* = .05) and BEScog (*r* = .203, *p* < .05), while negative associations were found with two of the alexithymia dimensions (TASif *r* = -.425, *p* = .001; TASdf *r* = -.334, *p* = .001), internalizing and externalizing psychopathology symptoms (ASRext *r* = -.261, *p* = .001; ASRint *r* = -.287, *p* = .001), and borderline traits (BPItot *r* = -.383, *p* = .001). For the RFQu, negative correlations were found with the Acting with awareness dimension of mindfulness (KIMSac *r* = -.313, *p* = .01), while positive associations were found with two dimensions of alexithymia (TASid *r* = .457, *p* = .001; TASdif *r* = .351, *p* = .001), internalizing and externalizing psychopathology symptoms (ASRext *r* = .349, *p* = .001; ASRint *r* = .218, *p* = .01) and borderline traits (BPItot *r* = .394, *p* = .001).

Except for BEScog and KIMSdes, all the correlations survived after Bonferroni corrections.

In contrast to findings in adults, no significant correlation was reported in adolescents between the RFQu subscale and the Describing dimension of mindfulness (KIMSdes *r* = -.176, *p* = .14), cognitive empathy (BEScog *r* = -.089, *p* = .32), and external-oriented thinking (TASeot *r* = .03, *p* = .80).

### Reflective functioning and NSSI

Thirty-three adolescents (16 females) and 51 adults (30 females) reported having engaged in at least one episode of NSSI in the previous year (see Table 5). Associations of recent NSSI with RFQc (χ^2^ = 5.07, *p* = .03, *ϕ* = -.144, *p* = .02) and RFQu (χ^2^ = 3.97, *p* = .04, *ϕ* = .128, *p* = .04) levels were observed in the adult sample, but not for the adolescent participants, although there was a trend in the same direction as in adults (certainty: χ^2^ = 2.06, *p* = .15, *ϕ* = -.131, *p* = .15; uncertainty: χ^2^ = 2.06, *p* = .15, *ϕ* = .132, *p* = .15).

**Table 5:** Descriptive statistics of the groups with and without Non-Suicidal Self-Injury (NSSI) and its association with reflective functioning in adolescence and adulthood.

Adults			
	With NSSI	Without NSSI	Group differences
% of total sample	20.81	79.19	
% of female	58.30	68.10	*p* = .233
Age	23.31	23.11	*p* = .642
% of high RFQc*	49.00	66.70	*p* = .02
% of high RFQu*	66.10	51.00	*p* = .04
Adolescents			
% of total sample	25.40	75.38	
% of female	51.50	51.00	*p* = .96
Age	15.59	15.97	*p* = .33
% of high RFQc	52.63	68.75	*p* = .15
% of high RFQu	64.21	53.13	*p* = .15

% of high RFQc = % of individuals with strong certainty about the links between mental states and actions

% of high RFQu = % of individuals with strong doubt about the link between mental states and actions; NSSI for Non Suicidal Self-Injury.

NSSI occurrence was missing for 1 adolescent and 8 adults.

*p < .05

## Discussion

The present study was designed to examine the psychometric properties of a French translation of the reflective functioning questionnaire in non-referred adolescent and adult samples. This study also aimed to gain more insight into the relationships between reflective functioning and non-suicidal self-injury in the community.

Confirmatory factor analyses showed measurement invariance across an adult and adolescent sample, as well as hypothesized patterns of associations with measures assessing concepts related to reflective functioning, such as mindfulness, alexithymia, empathy and perspective taking, and indices of clinical distress. In both the adolescent and the adult sample, satisfactory internal consistency for the “certainty about mental states” subscale and, to a slightly lesser extent, for the “uncertainty about mental states” subscale, was obtained. Given the small number of items in the questionnaire, the mean inter-item correlations were also considered. For both subscales, the coefficients were between r = .20 and r = .40, which is the range recommended by Briggs and Cheek [49] for adequate item homogeneity.

Although results for the two samples were broadly similar, two differences emerged between both samples. First, in adolescents, no association was found between the externally oriented thoughts subscale of alexithymia questionnaire and either of the two reflective functioning questionnaire subscales. The second difference concerns the relationships between the reflective functioning questionnaire uncertainty subscale, the describing dimension of mindfulness and the cognitive facet of empathy, which were significant in the adult sample, but not in the adolescents. Methodological factors (e.g., a lack of power in the adolescent group) may account for these differences. Problems with the validity of the externally oriented thought subscale of the alexythimia measure have been repeatedly demonstrated. Indeed, Zimmermann and colleagues [36] report a weak homogeneity specific to this dimension, with a Cronbach’s alpha of 0.47 and a mean inter-item correlation of 0.09. Finally, the multiple developmental changes that are inherent to adolescence may lead to a greater heterogeneity compared to the adult sample that could in turn influence the pattern of observed correlations. Further studies are needed to replicate the present results in a larger sample and investigate potential age differences in the relationship between cognitive empathy and reflective functioning. Nevertheless, given the configural invariance, satisfactory homogeneity indices and the finding that most of relationships with other constructs are similar in the adult and adolescent sample, overall results provide some confidence in the reliability and validity of the French version of the reflective functioning questionnaire.

Finally, the present results suggest that hypomentalizing (as expressed in low certainty and high uncertainty about mental states) may be associated with a recent history of episodes in adolescents and adults. In the adult sample, we found a significant association between a lower degree of certainty as well as a higher degree of uncertainty about mental states and a history of recent non-suicidal self-injury. Although no studies have directly addressed the relationship between non-suicidal self-injury and reflective functioning, dysfunctions in reflective functioning have been consistently highlighted in borderline personality disorder and allied conditions (see [30–31] for reviews). In particular, the present study supports, on the one hand, that a certain degree of certainty about the mental origin of behaviors may sustain an adaptive functioning as emphasized in the correlational pattern observed between the reflective functioning questionnaire certainty score and several measures of clinical and psychological variables. On the other hand, the associations found between reflective functioning and non-suicidal self-injury further suggest that an absence of this adaptive certainty about mental states concerning oneself and others (i.e. hypomentalizing reflected in certainty and high uncertainty scores) may characterize people who may be more prone to engage in non-suicidal self-injury. One possible interpretation of this finding relates to the major function of non-suicidal self-injury, namely, to regulate strong and overwhelming emotions to produce fast, but short-term, relief. This kind of body-centred self-regulation strategy may be indicative of disrupted RF abilities (hypomentalization) that prevent individuals from envisioning less action-based coping strategies. Conversely, higher reflective functioning may promote more mentalized, cognitive-based coping strategies, enabling the individual to regulate the arousal induced by events they encounter in daily life. This is consistent with the conceptualization of reflective functioning as being involved in resilience [50]. A calibrated degree of reflective functioning is indeed adaptive and may sustain the ability to revise past events, to consider others’ motives and feelings in the here and now, and to envision the future, thereby buffering the impact of adversity [50].

The situation might be slightly different for adolescents, in that similar trends but no significant associations were observed between features of reflective functioning and non-suicidal self-injury history. At least two hypotheses may account for these differences. As mentioned earlier, the adolescent sample may have been too small for statistically significant differences to be detected. As well as methodological issues, the present results may indicate underlying but partially distinct processes of non-suicidal self-injury according to the developmental period in which they occur. Non-suicidal self-injury consists of a wide range of heterogeneous behaviors that may vary in terms of type, function, and psychological or clinical correlates (see [51] for a review). In particular, differences have been observed between episodic and repetitive non-suicidal self-injury, including differences at the level of associated risk factors [52–54]. Episodic non-suicidal self-injury is by definition a transient phenomenon that typically has onset early in adolescence (12–14 years), while some epidemiological studies suggest that there is another peak at around 18–19 years [55]. While the prospective data on this issue remain scarce, on the basis of prevalence data, episodic non-suicidal self-injury is thought to be less frequent in adulthood [56]. In contrast to adolescents, recent episodes of non-suicidal self-injury reported by the adult group in the present study are therefore more likely to reflect chronic rather than acute manifestations of self-harming behavior. As previously illustrated with clinical correlates (e.g., [57]), our pattern of associations supports the suggestion that chronicity goes along with a more compromised profile of social cognition abilities. Thus, if the non significant relationship between reflective functioning and non-suicidal self-injury found in the adolescent sample is not due to methodological issues, the mechanism that underlies the distinct associations might not reflect a developmental shift resulting from age *per se*, but differences in the nature (occasional vs. repetitive) of the non-suicidal self-injury behaviors that are reported by adolescents compared to adults. The nature of self-harming behaviors reported in this sample of adolescents may be more transient than those reported by adults, which would weaken the relationship with uncertainty about mental states. Nevertheless, these conclusions remain speculative and need to be confirmed by a more comprehensive assessment of NSSI that includes a direct measure of chronicity.

Some limitations of the present study must be acknowledged. First, the results need to be replicated within clinical samples. The second limitation concerns the relatively small number of adolescents and adults reporting a recent history of non-suicidal self-injury. Further, the prevalence of NSSI in the present sample is very similar to lifetime prevalence in several recent studies (e.g., [17],). Hence, it is possible that participants, rather than considering the one-year occurrence of NSSI mentioned in the instructions of the BPI, reported on the lifetime incidence.

Indeed, NSSIs comprise heterogeneous phenomena, sometimes associated with various psychopathological symptoms, and their measurement can also vary following the methods that are employed. Their experiential and functional characteristics may also play a role in their variability from one sample to another. These clinical and qualitative features are likely to affect the potential relationships with RF processes such as those evaluated in the present study. Future investigation of this topic would definitely benefit from a more comprehensive evaluation of NSSI. The extent to which RF will be identified as a factor that underlies different types of self-harm remains an empirical question, for which the current results foster further inquiry.

In conclusion, the present study provides preliminary evidence for the reliability and validity of a self-report questionnaire to assess reflective functioning in French-speaking individuals and furthers our knowledge about individual differences in reflective functioning. The current data also constitute the first step towards the examination of reflective functioning in clinical settings of French-speaking population. Because these abilities seem to be impaired in several types of psychopathology, and because they might represent a putative mechanism of change common to different psychotherapeutic approaches [58], a reliable means of monitoring patients’ reflective functioning capacities would represent a step forward in the clinical practice of mental health professionals.

## References

[1] Fonagy P, Gergely G, Jurist EL, Target M (2002) Affect regulation mentalization and the development of the self New York: Other Press.

[2] Gergely G, Watson JS (1996) The social biofeedback theory of parental affect mirroring: the development of emotional self-awareness and self-control in infancy. Int J Psychoanal 77: 1181–1212. 9119582

[3] Fonagy P, Target M (1996) Playing with reality: Theory of mind and the normal development of psychic reality. Int J Psycho-Anal 77: 217–233. 8771375

[4] Slade A, Grienenberger J, Bernbach E, Levy D, Locker A (2005) Maternal reflective functioning attachment and the transmission gap: A preliminary study. Attachment Human Development 7: 283–298. 16210240 10.1080/14616730500245880

[5] Grienenberger J, Kelly K, Slade A (2005) Maternal reflective functioning mother–infant affective communication and infant attachment: Exploring the link between mental states and observed caregiving behavior in the intergenerational transmission of attachment. Attach Hum Dev 7: 299–311. 16210241 10.1080/14616730500245963

[6] Benbassat N, Priel B (2012) Parenting and adolescent adjustment: The role of parental reflective function. J Adolesc 35: 163–174. 10.1016/j.adolescence.2011.03.004 21497896

[7] Katznelson H (2014) Reflective functioning: A review. Clin Psychol Rev 34: 107–117. 10.1016/j.cpr.2013.12.003 24486522

[8] Bateman A, Fonagy P (2012) Handbook of mentalizing in mental health practice Washington: American Psychiatric Publishing.

[9] Ha C, Sharp C, Ensink K, Fonagy P, Cirino P (2013) The measurement of reflective function in adolescents with and without borderline traits. J Adolesc 36: 1215–1223. 10.1016/j.adolescence.2013.09.008 24215968

[10] Sharp C, Pane H, Ha C, Venta A, Patel AB, Sturek J, et al (2011) Theory of mind and emotion regulation difficulties in adolescents with borderline traits. J Am Acad Child Adolesc Psychiatry 50: 563–573. 10.1016/j.jaac.2011.01.017 21621140

[11] Taubner S, Curth C (2013) Mentalization mediates the relation between traumatic experiences and aggressive behavior in adolescence. Psihologija 46: 177–192.

[12] Taubner S, White LO, Zimmermann J, Fonagy P, Nolte T (2013) Attachment- related mentalization moderates the relationship between psychopathic traits and proactive aggression in adolescence. J Abnorm Child Psychol 41: 929–938. 10.1007/s10802-013-9736-x 23512713

[13] Karlsson R, Kermott A (2006) Reflective-functioning during the process in brief psychotherapies. Psychotherapy 43: 65–84. 10.1037/0033-3204.43.1.65 22121960

[14] Taubner S, Kessler H, Buchheim A, Kächele H, Staun L (2011) The role of mentalization in the psychoanalytic treatment of chronic depression. Psychiatry 74: 49–57. 10.1521/psyc.2011.74.1.49 21463170

[15] Levy K, Meehan KB, Kelly KM, Reynoso JS, Weber M, Clarkin JF, et al (2006) Change in attachment patterns and reflective function in a randomized control trial of transference-focused psychotherapy for borderline personality disorder. J Consult Clin Psychol 74: 1027–1040. 17154733 10.1037/0022-006X.74.6.1027

[16] Vermote R, Lowyck B, Luyten P, Vertommen H, Corveleyn J, Verhaest Y, et al (2010) Process and outcome in psychodynamic hospitalization-based treatment for patients with a personality disorder. J Nerv Ment Dis 198: 110–115. 10.1097/NMD.0b013e3181cc0d59 20145485

[17] Swannell SV, Martin GE, Page A, Hasking P, St John NJ (2014) Prevalence of Nonsuicidal Self-Injury in Nonclinical Samples: Systematic Review Meta-Analysis and MetaRegression. Suicide Life Threat Behav 44: 273–303. 10.1111/sltb.12070 24422986

[18] Brunner R, Kaess M, Parzer P, Fischer G, Carli V, Howen CV, et al (2013). Life-time prevalence and psychosocial correlates of adolescent direct self-injurious behavior: A comparative study of findings in 11 European countries. J Child Psychol Psychiatry 55: 337–348. 10.1111/jcpp.12166 24215434

[19] Zanarini MC, Frankenburg FR, Ridolfi ME, Jager-Hyman S, Hennen J, Gunderson JG (2006) Reported childhood onset of self- mutilation among borderline patients. J Personal Disord 20: 9–15. 10.1521/pedi.2006.20.1.9 16563075

[20] Chanen A, Kaess M (2012) Developmental Pathways to Borderline Personality Disorder. Curr Psychiatry Rep 14: 45–53. 10.1007/s11920-011-0242-y 22009682

[21] Fonagy P, Target M, Steele H, Steele M (1998) Reflective-Functioning manual: Version 5 for application to adult attachment interviews Unpublished manual London: University College.

[22] George C, Kaplan N, Main M (1985) Adult attachment interview Unpublished manuscript Berkeley: University of California.

[23] Target M, Oandasan C, Ensink K (2001) Child Reflective Functioning Scale scoring manual: For application to the child attachment Interview Unpublished manuscript UK: Anna Freud Centre/University College London.

[24] Ensink K (2004) Assessing theory of mind affective understanding and reflective functioning in primary school-aged children. PhD dissertation UK: University of London.

[25] Choi-Kain LW, Gunderson JG (2008) Mentalization: ontogeny assessment and application in the treatment of borderline personality disorder. Am J Psychiatry 165: 1127–1135. 10.1176/appi.ajp.2008.07081360 18676591

[26] Fonagy P, Luyten P, Moulton-Perkins A, Lee YW, Warren F, Howard S, et al. (2015) Development and Validation of a Self-report Measure of Mentalizing: The Reflective Functioning Questionnaire. Manuscript submitted for publication. 10.1371/journal.pone.0158678 PMC4938585 27392018

[27] Perkins A (2009) Feelings faces and food: Mentalization in borderline personality disorder and eating disorders. Dissertation Guildford UK: Department of Psychology University of Surrey.

[28] Perkins A (2011 May) Development and validation of a new self-report measure of mentalization: The 54-item Reflective Function Questionnaire. Presented at the SPR/BACP Conference Liverpool UK.

[29] Fonagy P, Leigh T, Steele M, Steele H, Kennedy R, Mattoon G, et al (1996) The relation of attachment status psychiatric classification and response to psychotherapy. J Consult Clin Psychol 64: 22–31. 8907081 10.1037//0022-006x.64.1.22

[30] Fonagy P, Luyten P (2009) A developmental mentalization-based approach to the understanding and treatment of borderline personality disorder. Dev Psychopathol 21: 1355–1381. 10.1017/S0954579409990198 19825272

[31] Herpertz SC, Bertsch K (2013) The social-cognitive basis of personality disorders. Curr Opin Psychiatry 27: 1–5. 10.1097/YCO.0000000000000026 24270477

[32] Jolliffe D, Farrington DP (2006) Development and validation of the Basic Empathy Scale. J Adolesc: 29 589–611. 16198409 10.1016/j.adolescence.2005.08.010

[33] D’Ambrosio F, Olivier M, Didon D, Besche C (2009) The basic empathy scale: A French validation of a measure of empathy in youth. Pers Individ Dif 46: 160–165.

[34] Bagby RM, Parker JDA, Taylor GJ (1994) The twenty-item Toronto Alexithymia Scale: Item selection and cross-validation of the factor structure. J Psychosom Res 38: 23–32. 8126686 10.1016/0022-3999(94)90005-1

[35] Loas G, Otmani O, Verrier A, Fremaux D, Marchand MP (1996) Factor analysis of the French version of the 20-item Toronto Alexithymia Scale (TAS-20). Psychopathology 29: 139–144. 8861519 10.1159/000284983

[36] Zimmermann G, Quartier V, Bernard M, Salamin V, Maggiori C (2007) Qualités psychométriques de la version française de la TAS-20 et prévalence de l’alexithymie chez 264 adolescents tout-venant. Encephale 33: 1–6. 18789786 10.1016/j.encep.2006.12.006

[37] Baer RA, Smith GT, Allen KB (2004) Assessment of mindfulness by self-report: the Kentucky inventory of mindfulness skills. Assessment 11: 191–206. 15358875 10.1177/1073191104268029

[38] Chabrol H, Montovany A, Ducongé E, Kallmeyer A, Mullet E, Leichsenrig F (2004) Factor structure of the borderline personality inventory in adolescent. Eur J Psychol Assess 20: 59–65.

[39] Leichsenring F (1999) Development and first results of the borderline personality inventory: A self-report instrument for assessing borderline personality organization. J Pers Assess 73: 45–63. 10497801 10.1207/S15327752JPA730104

[40] Achenbach TM, Rescorla LA. Manual for the ASEBA adult forms and profiles Burlington: University of Vermont, 61.

[41] Wyss CA, Voelker SL, Cornok BL, Hakim-Larson J (2003) Psychometric properties of a French-Canadian translation of Achenbach’s youth self-report. Can J Behav Sci 35: 67–71.

[42] Muthén LK, Muthén BO (2006) Mplus user's guide Los Angeles: Muthén Muthén.

[43] Beaton D, Bombardier C, Guillemin F (2002) Recommendations for the cross-cultural adaptation of health status measures New York: American Academy of Orthopaedic Surgeons Institute for Work and Health.

[44] Heene H, Hilbert S, Freudenthaler H, Bühner M (2012) Sensitivity of SEM fit indexes with respect to violation of uncorrelated errors. Struct Equ Modeling 19 : 36–50.

[45] Brown TA (2006) Confirmatory factor analysis for applied research New York: Guilford Press.

[46] Hu LT, Bentler PM (1999) Cut off criteria for fit indexes in covariance structure analysis: Conventional criteria versus new alternatives. Struct Equ Modeling 6: 1–55.

[47] Yu CY (2002) Evaluating cutoff criteria of model fit indices for latent variable models with binary and continuous outcomes. Dissertation Los Angeles: University of California.

[48] Byrne BM, Stewart SM (2006) The MACS approach to testing for multigroup invariance of a second-order structure: a walk through the process. Struct Equ Modeling 13: 287–321.

[49] Briggs SR, Cheek JM (1986) The role of factor analysis in the evaluation of personality scales. J Pers 54: 106–148.

[50] Allen JG, Fonagy P (2006) Handbook of mentalization-based treatment Chichester: John Wiley.

[51] Klonsky ED, Muehlenkamp JJ (2007) Self‐injury: A research review for the practitioner. J Clin Psychol 63: 1045–1056. 17932985 10.1002/jclp.20412

[52] Brunner R, Parzer P, Haffner J, Steen R, Roos J, Klett M, et al (2007) Prevalence and psychological correlates of occasional and repetitive deliberate self-harm in adolescents. Arch Pediatr Adolesc Med 161: 641–649. 17606826 10.1001/archpedi.161.7.641

[53] Hawton K, Rodham K, Evans E, Weatherall R (2002) Deliberate self harm in adolescents: self report survey in schools in England. Br Med J 325: 1207–11. 12446536 10.1136/bmj.325.7374.1207 PMC135492

[54] Kakhnovets R, Young HL, Purnell A, Huebner E, Bishop C. (2010) Self-reported experience of self injurious behavior in college students. J Mental Health Counseling 32: 309–323.

[55] Kerr PL, Muehlenkamp JJ, Turner JM (2010) Nonsuicidal self-injury: a review of current research for family medicine and primary care physicians. J Am Board Fam Med 23: 240–259. 10.3122/jabfm.2010.02.090110 20207935

[56] Whitlock JL, Selekman M (in press) Non-suicidal self-injury (NSSI) across thelifespan.

[57] Manca M, Presaghi F, Cerutti R (2014) Clinical specificity of acute versus chronic self-injury: Measurement and evaluation of repetitive non-suicidal self-injury Psychiatry Res 215: 111–119. 10.1016/j.psychres.2013.10.010 24210667

[58] Fonagy P, Allison (2014) The role of mentalizing and epistemic trust in the therapeutic relationship. Psychotherapy 51: 372–280. 10.1037/a0036505 24773092

